# Quantitative Structure‐activity Relationship (QSAR) Models for Docking Score Correction

**DOI:** 10.1002/minf.201600013

**Published:** 2016-04-29

**Authors:** Yoshifumi Fukunishi, Satoshi Yamasaki, Isao Yasumatsu, Koh Takeuchi, Takashi Kurosawa, Haruki Nakamura

**Affiliations:** ^1^ Molecular Profiling Research Center for Drug Discovery (molprof)National Institute of Advanced Industrial Science and Technology (AIST)2-3-26, AomiKoto-ku, Tokyo135-0064Japan; ^2^ Technology Research Association for Next-Generation Natural Products Chemistry2-3-26, AomiKoto-ku, Tokyo135-0064Japan; ^3^ Daiichi Sankyo RD Novare Co., Ltd.1-16-13, Kita-KasaiEdogawa-ku, Tokyo134-8630Japan; ^4^ Hitachi Solutions East Japan12-1 EkimaehonchoKawasaki-ku, Kanagawa210-0007Japan; ^5^ Institute for Protein ResearchOsaka University3-2 YamadaokaSuita, Osaka565-0871Japan

**Keywords:** Binding free energy, ChEMBL, Docking score, Protein-compound docking

## Abstract

In order to improve docking score correction, we developed several structure‐based quantitative structure activity relationship (QSAR) models by protein‐drug docking simulations and applied these models to public affinity data. The prediction models used descriptor‐based regression, and the compound descriptor was a set of docking scores against multiple (∼600) proteins including nontargets. The binding free energy that corresponded to the docking score was approximated by a weighted average of docking scores for multiple proteins, and we tried linear, weighted linear and polynomial regression models considering the compound similarities. In addition, we tried a combination of these regression models for individual data sets such as IC_50_, K_i_, and %inhibition values. The cross‐validation results showed that the weighted linear model was more accurate than the simple linear regression model. Thus, the QSAR approaches based on the affinity data of public databases should improve docking scores.

## 1 Introduction

De novo drug design is a key factor in lead optimizations and the selective optimization of side activities, and the quantitative structure‐activity relationship (QSAR) approach is a useful tool for predicting target/off‐target activities. QSAR‐based affinity predictions are useful for the drug repositioning (drug repurposing) of known approved drugs, poly‐pharmacology and the prediction of drug−drug interactions.^1^, ^2^, ^3^, ^4^, ^5^, ^6^, ^7^, ^8^, ^9^, ^10^, ^11^, ^12^, ^13^, ^14^, ^15^, ^16^, ^17^, ^18^, ^19^, ^20^, ^21^, ^22^ hERG‐inhibition and cytochrome P450 (CYP)‐inhibition predictions represent classical achievements in off‐target predictions, and this kind of target/off‐target prediction is known as counter screening. The recent accumulation of protein‐compound affinity data in public repositories, such as the PubChem and ChEMBL projects, has enabled us to carry out proteome‐wide target/off‐target predictions.^23^,^24^ These predictions are based on structure‐activity relationship models for multiple proteins, just as in the conventional computer‐aided drug design and virtual screening.

In a previous study, we attempted affinity and target predictions of a compound by using docking studies against multiple proteins. These trials worked well in virtual screenings. However, these methods provided only binary, active/inactive information and could not provide quantitative affinities.^6^,^9^,^10^ Target/off‐target predictions based on QSAR and counter screening have succeeded in many studies, including ours.^4^,^5^,^7^,^8^,^11^, ^12^, ^13^, ^14^, ^15^, ^16^, ^17^, ^18^, ^19^, ^20^, ^21^, ^22^ However, the somewhat primitive approach of a similarity search relying on the QSAR model is not sufficiently versatile. To broaden its applicability, the similarity search has been improved by considering the similarity that is shared among different groups of compounds rather than the similarity between two compounds.^11^ Most QSAR models rely on descriptors with sets of two‐dimensional (2D) substructures; the most popular such descriptors are the MDL's MACCS key and Dragon‐X (Talete srl, Milano, Italy). Moreover, there have been many regression models. In our previous studies, we used a protein‐compound affinity matrix as the set of descriptors and successfully predicted CYP inhibitors and substrates.^7^,^12^

As an extension of our previous work,^6^,^7^ in the present study we developed and examined some principal component regression‐type prediction methods based on the machine‐learning score modification (MSM) method and the docking score index (DSI: protein‐compound affinity matrix) using public repository data. In the MSM method, the docking score against a target protein could be corrected by a linear combination of docking scores against multiple nontarget proteins. The DSI of a compound is a set of docking scores of the compound against multiple target and nontarget proteins. Here, the nontarget proteins are used as “probes” to check the three‐dimensional (3D) shape and distribution of the atomic charge of the compound. We applied our methods to kinases of the ChEMBL database, because kinases form a major protein family that is key to understanding and controlling cellular signal transduction, and because their structures have been studied thoroughly.^25^, ^26^, ^27^

## 2 Methods

### 2.1 Prediction Models

The present method predicts the binding energy (affinity) of given protein‐compound pairs based on the protein‐compound docking scores obtained by a docking program; this method is a descriptor‐based machine‐learning or regression method.^6^,^7^,^28^,^29^ The present method requires a learning set of 3D structures of compounds, the binding energy data between those compounds, and target proteins. Let *s_i_* ^*b*^, *R_b_* ^*a*^, and *β* be the docking score of the *i*‐th compound, that of the *b*‐th protein, and parameters, respectively. The set of {b} can include the target protein (the *a*‐th protein). We proposed a score modification method as follows. (Eq. (1))^6^

(1) $$\Delta G_{i}^{a}=\sum_{b=1}s_{i}^{b}\cdot R_{b}^{a}+\beta$$

Here, *ΔG_i_* ^*a*^ is the binding free energy between the *i*‐th compound and the *a*‐th protein. Proteins that are similar to the target protein could bind the ligands of the target protein. Docking scores correspond to the binding free energy, and the ensemble average should improve the accuracy. Thus eq. 1 should work, and indeed, this approximation was successfully applied to several targets.^6^,^7^,^28^,^29^ The score modification method increased the area under the curve (AUC) values of the database enrichment curves by 50 %. Namely, the AUC values of 60–70 % were improved to 80–90 % in these previous reports. In addition, there is one advantage to the other conventional QSAR models with ordinary molecular descriptors. One of the most serious problems of QSAR models is the limited range of applicable domains, since QSAR models cannot work for unexpected input data.^30^ If the docking score is precisely proportional to the binding free energy without computational error, *ΔG_i_^a^=s_i_* ^*a*^. Thus, eq. 1 can work without any experimental affinity data and the problem of identifying an applicable domain is avoided.

In eq. 1, the number of parameters is equal to the number of proteins. The number of parameters can be reduced by principal component regression (PCR). Docking scores should be a form of some kinds of similarity scores (see APPENDIX A). Thus, the docking scores could be used as the descriptors for compounds. If we allow docking scores as descriptors, the docking score for a target protein is not needed in eq. 1.

**Figure 1:**
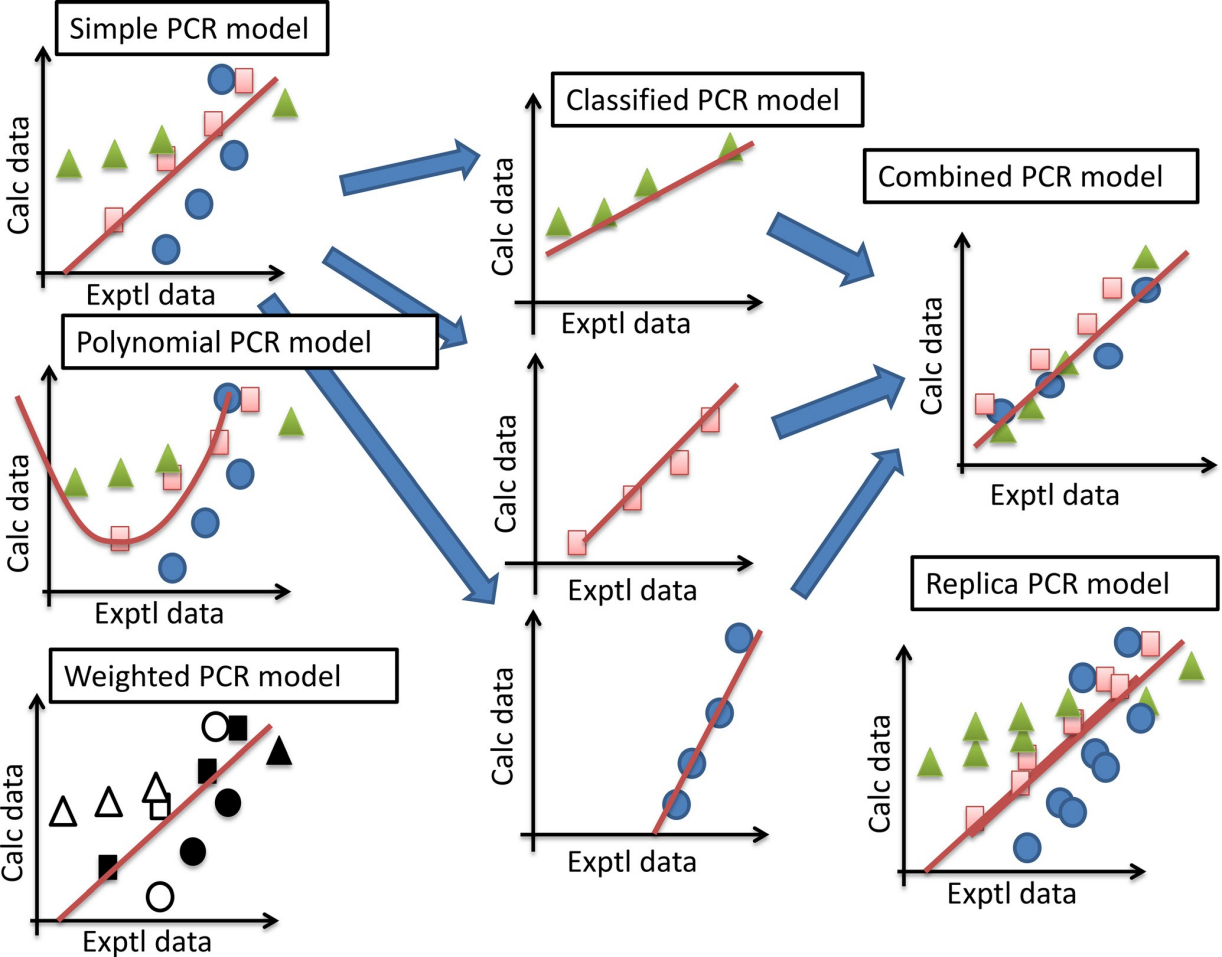
Schematic representation of each principal component regression (PCR) model.

In the present model, the protein‐compound binding energy *ΔG_i_* ^*a*^ is approximated by the PCR method based on the protein‐compound docking scores *s_i_* ^*b*^. As shown in Figure 1, we tried six PCR models (Models 1–6) as follows. In each model, the optimal principal component analysis (PCA) axis was selected to maximize the q value by the leave‐one‐out (LOO) cross‐validation test. The selection of the PCA axis corresponds to the factor rotation.^29^

(Model 1) Linear PCR model (Eq. (2), (3))

(2) $$\Delta G_{i}^{a}=\sum_{j=1}^{N}c_{j}^{a}\cdot p_{i}^{j}+b$$

(3) $$p_{i}^{j}=\sum_{b=1}^{Np}d_{b}^{j}\cdot \left(s_{i}^{b}-<s^{b}>\right)$$

Here, *c_j_* ^*a*^, *b*, *p*, and *d_b_* ^*j*^ are the parameter, offset parameter, principal component vector, and loading vector, respectively. The ⟨ ⟩ represents an average. The PCA of the protein‐compound docking score matrix *s* gives the loading vector *d* and the principal component vector (axis) *p*. The parameters *c* and *b* are determined by a multilinear regression (MLR). *N_p_* is the total number of docked proteins and *N* (*N*<*N_p_*) is determined to maximize the *q*‐value obtained by the LOO cross‐validations. The parameters are determined based on the learning set and then are used for prediction.

The protein‐compound docking scores *s_i_* ^*b*^ were obtained by the program Sievgene,^31^ which is a protein‐ligand flexible docking program for *in silico* drug screening. Sievgene is a part of the myPresto system, which is available online (http://presto.protein.osaka‐u.ac.jp/myPresto4/) and is free for academic use.

(Model 2) Polynomial PCR model (Eq. (4), (5))

(4) $$\Delta G_{i}^{a}=\sum_{j=1}^{N}c_{j}^{a}\cdot p_{i}^{j}+\sum_{k=1}^{N}\sum_{j\neq k}^{N}c2_{j}^{a}\cdot p_{i}^{j}\cdot p_{i}^{k}+b$$

(5) $$p_{i}^{j}=\sum_{b=1}^{Np}d_{b}^{j}\cdot \left(s_{i}^{b}-<s^{b}>\right)$$

Here, *c2_j_* ^*a*^ represents the parameter for the second‐order term. We tried only the second‐order and did not try the higher‐order polynomials. The other terms and parameters are defined exactly as in Model 1.

(Model 3) Weighted learning PCR model. (Eq. (6))

(6) $$S=\sum_{b=1}^{Np}{\left(s_{i}^{b}-s_{j}^{b}\right)}^{2}$$

*S* is the distance between the *i*‐th and *j*‐th compounds. Let S_max_(*i*)=max{S; for all *j*}. The data of compounds that satisfy *S*<*x* ⋅ S_max_ are replicated *M* times. In the present study, *x*=0.1, 0.2, 0.3, and 0.5 were examined and *M* was set to 1, 2, 4, and 8.

(Model 4) Classified PCR model

In the classified PCR model, the experimental data are classified into *IC_50_*, *K_i_*, and %inhibition data, and the simple PCR method is applied to each classified data set.

(Model 5) Combined PCR model

This model is a linear combination of the regression models made by the classified PCR model. (Eq. (7))

(7) $$\Delta G_{i}^{a}=\sum_{m=1}^{Ntype}C_{m}\cdot \Delta g_{m}^{a}+c_{0}$$

Here, *Δg*, *C_m_* and *c_0_* are the binding free energy obtained from the *m*‐th data set and the fitting parameters, respectively. The *Δg* is given by eq. 1, and the coefficient *C_m_* is determined to minimize the root‐mean‐square difference between the coefficients of {c} of *Δg*. In the present study, the experimental data were classified into *IC_50_*, *K_i_*, and %inhibition data (*m*=*K_i_*, *IC_50_* and %inhibition). We based this classification on the individual source of the experimental data.

(Model 6) Replica and partial‐replica PCR models

Cortes‐Ciriano *et al*. suggested that the use of multiple replica data sets permutated by random noise could improve the QSAR accuracy.^32^ In the replica PCR method, the experimental data and docking scores are replicated by the permutation of 5 % noise. Also, Steinmetz *et al*. suggested that the QSAR result could be improved by considering the importance of data due to reliability.^33^ In the partial‐replica PCR method, experimental *ΔG* values <−10 kcal/mol are replicated by permutation of 2 % noise, since the strong affinities of lead‐level compounds are observed by multiple experiments in many cases and should be more reliable than the weak affinities of hit‐level compounds.

### 2.2 Generation of the Docking Score Index by Protein‐compound Docking

The protein‐compound docking scores *s_i_* ^*b*^ in all models were calculated by the protein‐compound docking program Sievgene.^31^ Sievgene generates multiple possible conformers for each compound and keeps the structures of target proteins more or less rigid, with the exception that soft interaction forces can change the structures slightly. This docking program reconstructed about 50 % of the receptor‐compound complexes in PDB (132 in total) with an accuracy of less than 2 Å root mean square deviation (RMSD),^31^ which is mostly equivalent to the predictions by other docking programs. In the present study, the Sievgene program generated up to 100 conformers for each compound, and 200×200×200 grid potentials were adapted for all proteins. The pocket regions were suggested by the coordinates of the original ligands in the receptor‐compound complex structures. The details of the docking score are summarized in Appendix B (Supporting Information). It takes 3 seconds to dock one compound against one protein on a single core of the Xeon 5570 CPU (2.98 GHz).

### 2.3 Data‐conversion Method

The protein‐compound binding energy *ΔG* is calculated from the *K_d_* value as follows: (Eq. (8))

(8) $$\Delta G=k_{B}\cdot T\cdot \ln\left(K_{d}\right)$$

where *k_B_* and *T* are the Boltzmann constant and temperature.

The experimental *K_d_* and *ΔG* values are difficult to obtain and quite rare in public databases. On the other hand, the %inhibition, *K_i_* and *IC_50_* values are relatively easy to obtain and abundant in public databases such as PubChem and ChEMBL. In the present study, we assumed that *K_d_*=*K_i_*, since the binding affinities of the natural ligands have been reported to be much weaker than those of the reported artificial ligands in many proteins. For the %inhibition and *IC_50_* data, the conventional approaches are adopted as follows. The %inhibition value is converted to the *K_i_* value. Let *E*, *S*, *P* and *I* be the enzyme, substrate, product and inhibitor, respectively. The inhibition reaction is described as follows. Here, “*K*” represents the reaction rate. (Eq. (9))

(9) $$E+S\overset{K_{s}}{\underset{}{↔}}ES\overset{K_{2}}{\underset{}{\to }}E+PE+I\overset{K_{i}}{\underset{}{↔}}EI$$

When the enzyme reaction is the rate‐determining step, we have (Eq. (10))

(10) ES→E+P.

The value of *K_s_* is then derived from the density of the *E*, *S* and *ES* complexes as follows. (Eq. (11))

(11) $$K_{s}=\frac{\left[E][S\right]}{\left[ES\right]}$$

Here, the bracket [ ] represents the density of molecules. The reaction speed *v* is described as follows. (Eq. (12))

(12) $$v=\frac{V_{\max}\left[S\right]}{\left(1+\left[I\right]/K_{i}\right)K_{s}+\left[S\right]}$$

Here, *V_max_* is the maximum enzyme reaction speed. Let *r* be the residual activity; *K_i_* is then given by (Eq. (13))

(13) $$K_{i}=\frac{\left[I\right]}{\frac{\left[S]/r-[S\right]}{K_{s}}-1}$$

The parameters in eq. 13 must satisfy (Eq. (14))

(14) $$r<\frac{\left[S\right]/K_{s}}{1+\left[S\right]/K_{s}},$$

because *K_i_*>0. Here, the %inhibition value is (1−*r*)*100.

The *IC_50_* value is converted to the *K_i_* value by the Cheng‐Prusoff equation as follows.^20^,^34^ Here, *S* and *K_s_* are the substrate and the affinity between the enzyme and the substrate. (Eq. (15))

(15) $$K_{i}=\frac{IC_{50}}{1+\frac{\left[S\right]}{K_{s}}}$$

Unfortunately, the exact values of [S], [I] and *K_s_* are not explicitly described in the assay data of PubChem or ChEMBL. Thus, we checked some original experimental articles for their assay data and adopted arbitrary standard values for [S], [I] and *K_s_* based on previous reports.^27^,^28^,^35^, ^36^, ^37^

## 3 Data Preparation

### 3.1 Probe Protein Sets with and without Kinase Structures

To generate {*s*} in Models 1–6 (the DSI or affinity fingerprint), we performed a protein−compound docking simulation based on the soluble protein structures registered in the Protein Data Bank (PDB). The probe protein set consisted of 600 arbitrarily selected protein structures. These structures were all protein‐ligand complex structures. Some of them were kinases. For protein sets, the complexes containing a covalent bond between the protein and ligand were removed, and all missing hydrogen atoms were added to form the all‐atom models of the proteins. All water molecules and cofactors were removed from the protein structures. The atomic charges of the proteins were the same as those in AMBER parm99.^38^ The docking pocket of each protein was indicated by the coordinates of the original ligand.

### 3.2 Validation Test Set: Target Proteins and Compounds

The tested compounds and their assay information (compound structures, affinities against kinases) were downloaded from KinaseSARfari on the ChEMBL website (https://www.ebi.ac.uk/chembl/).^24^ The biochemical assay data, namely, *K_i_*, *IC_50_*, %residual activity and/or %inhibition values of human kinase protein‐inhibitor systems, were also extracted from the bioactivity table in KinaseSARfari. Assay data with inadequate energy units or unclear energy values were excluded. The assay data for large compounds (mass weight >500 Da) were also excluded, since Sievgene is designed for the docking of small compounds with mass weights <500 Da.

As target proteins, 97 kinases with more than 50 assay data points were selected. The 3D structures of the compounds were energy‐optimized by cosgene^39^ with the general AMBER force field (GAFF),^40^ and the atomic charges were calculated by the MOPAC AM1 model using the Hgene program of the myPresto suite. Finally, 38,946 assay data points of 97 kinases and 18,491 compounds were derived. Most of the assay data were *IC*_50_ data, with the second‐most common type being %inhibition data, followed by *K_i_* and *K_d_* values. These data were converted to the *ΔG* value by using Eqs. 8, 13 and 15. Equations 13 and 15 required the density of the substrate [*S*], the density of the inhibitor [I], and the reaction rate of the substrate (*K_s_*). These [*S*], [*I*] and *K_s_* data were not available in the ChEMBL database. For Eq. 15, we used the [*S*] and *K_s_* values reported by Carna Bioscience Inc. (http://www.carnabio.com/english/), and we used the standard values ([*S*] : *K_s_*=1 : 1) in place of unknown data. For Eq. 13, we used constant values for some parameters: [*S*]=20 μM, [*I*]=50 μM and *K_s_*=1 μM. And *r* values greater than 0.95 and less than 0.05 were ignored, since the error of *r* should be around 5 % and an *r* value >1 gave unreasonable *ΔG* values. The parameter set was determined based on several reports of assays included in the ChEMBL database.

## 4 Results and Discussion

### 4.1 *ΔG* Values Obtained from *K_i_*, *IC_50_* and %Inhibition Values

Appendix C (Supporting Information) provides a list of the target kinases used in the present study and the number of ligands for each. The kinase names were the domain names from the KinaseSARfari database in ChEMBL.

The average and standard deviation values of the experimental *ΔG* values of the 97 target proteins are summarized in Table 1, and the data were classified following the data type. For some kinases, multiple experimental assay data (*K_i_*, *IC_50_* and %inhibition) were available for the same ligands of some proteins. The differences between each classified data type (the *ΔG* value calculated from *K_i_*, *IC_50_* and %inhibition data) and the other types of data are summarized in Table 1. The difference corresponded to the error of the experimental data converted in the present study by Eqs. 13 and 15.

**Table 1:** Statistics of *ΔG* values (kcal/mol) converted from ChEMBL data.

Data type	Average *ΔG*	σ [a]	ΔGmin [b]	ΔGmax [c]	RMSD [d]
Whole	−9.67	2.41	−18.51	−0.55	–
Ki/Kd	−9.30	1.69	−16.30	−1.34	1.55
IC_50_	−9.37	2.18	−18.51	−0.55	1.87
Activity	−0.88	5.74	−9.35	−4.11	2.58
%inhibition	−2.66	4.14	−9.35	−4.11	2.99

[a] The standard deviation of the whole observed data (kcal/mol). [b] The minimum ΔG value of the data set (kcal/mol). [c] The maximum ΔG value of the data set (kcal/mol). [d] The root mean square deviation (RMSD) of the multiply‐observed data for the same protein‐ligand pairs (kcal/mol)

We simulated the expected correlation coefficient between the experimental and calculated *ΔG* values based on the statistics summarized in Table 1. We generated a set of numbers that mimics the experimental *ΔG* values whose average and standard deviation were −9.6 kcal/mol and 2.5 kcal/mol, respectively. Then a random number was added as experimental error to each simulated experimental *ΔG* value. Also, we generated a set of numbers that mimics the calculated *ΔG* value by adding a random number as the computational error. The calculated correlation coefficients are summarized in Table 2. In addition, we performed a set of virtual screenings based on these simulated data. The compounds with experimental *ΔG*<−11 kcal/mol were selected as the active (hit) compounds, and the others were treated as decoys. Then the compounds were sorted in the order of the calculated *ΔG* and the receiver operating characteristic (ROC) curves were calculated. The area‐under‐the‐curve (AUC) values of the ROC curves of the simulated virtual screenings are also summarized in Table 2. A higher AUC value corresponds to better prediction, and the AUC value is always more than zero and less than 100 %. For the random screening, AUC=50 %.

**Table 2:** Correlation coefficient (R) and area under the curve (AUC) of the receiver operating characteristic (ROC) curve by mathematical simulation.

Error exp [a]	Error calc [b]	R	AUC (%)
0.58	0.82	0.90	97
0.58	1.29	0.79	94
0.58	1.51	0.74	91
0.58	1.83	0.67	88
0.70	0.91	0.88	97
0.70	1.35	0.78	94
0.70	1.55	0.73	92
0.70	1.87	0.66	88
1.16	1.30	0.79	97
1.16	1.64	0.70	93
1.16	1.81	0.66	92
1.16	2.09	0.59	88
1.39	1.51	0.75	97
1.39	1.81	0.66	94
1.39	1.97	0.62	92
1.39	2.23	0.56	88

[a] Simulated experimental error (kcal/mol). [b] Simulated prediction error (kcal/mol).

These correlation coefficients (0.6–0.7) suggested poor QSARs; on the other hand, the AUC values (AUC=80–90 %) showed good virtual screening results.

### 4.2 Cross‐validation Tests of QSAR Models

**Figure 2:**
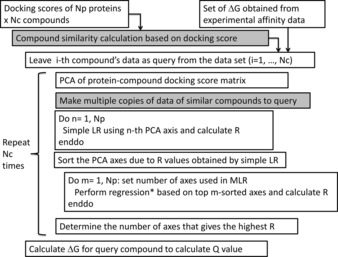
Schematic representation of the leave‐one‐out (LOO) cross‐validation procedure for models 1–6. The similarity calculation and making copy of assay data (in model 6) were applied only for the weighted principle component regression (PCR) model (gray boxes). *Nc* is the number of compounds. For the replica and partial‐replica PCR models, the initial docking scores and set of *ΔG* were replicated. *: Regression type was polynomial only for the polynomial PCR model. Otherwise, the regression was a multilinear regression (MLR).

Each of the compounds that gave assay data for one or more of the 97 target proteins was docked to all proteins of a protein set to generate the protein‐compound docking score matrix *s*. Then we adopted Models 1–6 and the LOO cross‐validation test to calculate the correlation coefficients R and Q. Figure 2 shows the schematic description of the LOO cross‐validation procedure for models 1–6. In all models, we performed the simple linear regressions and obtained an R value for each principal component axis. The axes were sorted according to their R values. Then, we performed the multiple linear/polynomial regressions adopting the top m‐axes and calculated the R values. The number of axes that gave the highest R was adopted to construct the regression model for calculating the *ΔG* of the query compound, and the Q value was calculated.

**Figure 3:**
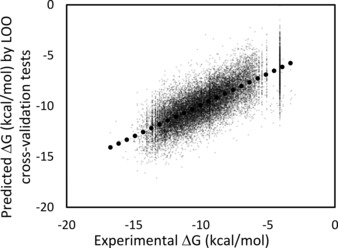
Correlation between the experimental and prediction data for all 97 kinase proteins obtained by the weighted PCR model with *x*=0.1 and the number of replicas=1. Black dots represent the least‐squares fitting line, and the correlation coefficient is 0.76. The dotted line represents the fitted result.

Figure 3 shows the results of the LOO cross‐validation test of three selected kinases. The correlation coefficients (R) between the experimental and predicted *ΔG* values are summarized in Tables 1 and 3, respectively. Also, the root mean square error (RMSE) values between the experimental and predicted *ΔG* values are summarized in Table 3.

**Table 3:** Average correlation coefficient (R/Q) between the experimental data and the calculated data obtained by the six regression models over all 79 proteins.

Model			R [a]	RMSE [b]	Q [c]	RMSE [d]
Simple PCR model			0.81	1.17	0.63	1.58
Polynomial PCR model			0.69	1.47	0.58	1.66
Replica PCR model	NR [e]					
	1		0.81	1.17	0.63	1.59
	2		0.81	1.17	0.63	1.59
	5		0.81	1.17	0.62	1.60
	10		0.81	1.17	0.60	1.64
Partial‐replica PCR model	NR [e]					
	1		0.82	1.19	0.63	1.59
	2		0.82	1.16	0.63	1.59
	5		0.82	3.26	0.62	1.60
	10		0.82	1.19	0.60	1.63
Weighted PCR model	x	NR [e]				
	0.1	1	0.89	0.87	0.66	1.54
		2	0.89	0.87	0.66	1.54
		4	0.89	0.87	0.66	1.54
		8	0.89	0.87	0.65	1.56
	0.2	1	0.89	0.87	0.66	1.54
		2	0.89	0.87	0.65	1.55
		4	0.89	0.87	0.65	1.55
		8	0.89	0.87	0.64	1.57
	0.3	1	0.89	0.87	0.65	1.54
		2	0.89	0.87	0.65	1.55
		4	0.89	0.87	0.65	1.56
		8	0.89	0.87	0.64	1.58
	0.5	1	0.89	0.87	0.65	1.54
		2	0.89	0.87	0.65	1.55
		4	0.89	0.87	0.64	1.56
		8	0.89	0.87	0.63	1.58
Classified PCR			0.92	0.42	0.71	0.98
Combined PCR			0.92	0.42	0.61 [f]	ND [f]

[a] Average correlation coefficient between the experimental and calculated data. [b] Average root mean square deviation (RMSD) error between the experimental and calculated data (kcal/mol). [c] Average correlation coefficient between the experimental and calculated data obtained by the leave‐one‐out (LOO) cross‐validation test. [d] Average RMSD error between the experimental and calculated data obtained by the LOO cross‐validation test (kcal/mol). [e] Number of replicas. [f] In 12 cases out of 97 proteins, the root mean square deviation error (RMSE)>10^6^.

The machine‐learning DSI and MSM methods were score modification methods in which the new docking scores were given by the linear combinations of the protein‐compound docking scores. The previous works reported the AUC values of database enrichment curves obtained by the machine‐learning DSI/MTS methods and the AUC values were about 98 % (in the original works, the AUC values were referred to as q values). When the number of active compounds is much smaller than the number of inactive compounds, the AUC of the database enrichment curve is close to the AUC of the ROC curve, and the data sets of the previous works satisfy the condition (number of actives : number of in‐actives=1 : 1000). Based on Table 2, an AUC of 98 % corresponded to an R of 0.8–0.9. The R values were close to the R values obtained by the weighted PCR models in Table 3. We must note that the data sets used in the previous studies consisted of high affinity compounds (e.g., commercial drugs) as active compounds and decoy compounds. In the present study, all compounds were nearly active, and discriminating between strong and weak active compounds is more difficult than distinguishing highly active and inactive compounds as in the previous reports. Thus, the present validation tests were much more strict than those used in the previous studies. In addition, the previous methods only realized the active/in‐active binary decision in virtual screening. On the other hand, the present methods could evaluate the binding energy value, which is essential in drug design.

The simple PCR model (Model 1) worked well, since the correlation coefficients between the experimental and calculated *ΔG* values were close to those obtained by the mathematical simulation data. Also, the results obtained by the simple PCR model were close to the averaged correlation coefficient and RMSE values obtained by the docking study (R=0.7 and 2–3 kcal/mol). Considering that the present model did not require the target protein structure, even the simple PCR model should be useful for rough affinity estimation in 21 % of cases in which Q>0.7 out of the 97 target proteins.

For the polynomial PCR model, the polynomial was restricted to the second order, since the high‐order polynomials require many parameters in the regression equation. This trial did not improve the correlation to the experimental data. This suggests that the linear model was sufficient in the present study.

Figure 3 shows the results obtained by the weighted PCR model. In the weighted PCR method, the molecule in the teaching data that was similar to the input molecule was copied multiple times. The similarity was calculated by the docking score in eq. 6, and *x*=0.1, 0.2, 0.3 and 0.5 were examined. The weighted PCR method with *x*=0.1 and single or double copies of similar compounds showed the best correlation to the experimental data. This method required the same data as the simple PCR model. In addition to our method, however, it is expected that many other approaches could be taken to generate the replica data. The weighted PCR model should be useful for rough affinity estimation among these models, and Q>0.7 in 37 % of cases in the present study. The simple average of Q over the 97 proteins in Table 2 was about 0.64, and the correlation coefficient of the *ΔG* value of the whole data was 0.76 (see Figure 3).

The experimental data were classified into *IC_50_*, *K_i_* and %inhibition data sets, and then the classified PCR method was applied to each. The correlations were improved compared to the other models with unclassified data. We must note that the Q values obtained by the classified PCR model cannot be simply compared to those obtained by the other models, since there were fewer classified experimental data than unclassified data.

The combined PCR method did not improve the correlation between the experimental and calculated *ΔG* values compared to the simple PCR model. In particular, in some cases, the combined PCR model generated unrealistic *Δ*G values with extreme computational error (>10^6^ kcal/mol). In 9 cases (9 %), the RMSE values obtained by the combined PCR model were <1 kcal/mol, while only three RMSE values obtained by the weighted PCR model were <1 kcal/mol (3 %). This result suggested that a deep learning method that considers the study from which the data was sourced should work better than the method employed in the present study, and that the data manipulation should be performed carefully while considering the applicable domain of the model.

The replica PCR and partial‐replica PCR models did not work in the present study. The greater the number of replicas, the lower the correlation coefficients became. Since this study was a single trial with simple random noise, the results do not contradict those of the previous works.^32^,^33^ The duplication of data should be treated more carefully than it is in the simple duplication.

## 5 Conclusions

In order to achieve docking score correction, we developed several QSAR models based on combinations of multiple docking scores by protein‐drug docking simulations and applied heterogeneous public data to these models. The prediction models employed a descriptor‐based PCR, and the compound descriptor was a set of docking scores against many nontarget proteins (DSI, protein‐compound affinity matrix or affinity fingerprint). We tried six variations of the PCR models: simple, polynomial, weighted, classified, replica PCR and combined PCR models.

Even the simple PCR model worked in some cases, but when the assay data were classified into *IC_50_*, *K_i_* and %inhibition data, the classified PCR model worked better than the simple PCR model. The linear combination of the QSAR models (combined PCR model) did not improve the results compared to the simple PCR. Although the weighted PCR model was simple, it achieved the same results as the more complex combined PCR model. In general, the weighted PCR model should be easier and more accurate than the other models. In cases in which the original sources of the assay data are easily accessible, the classified/combined PCR models could be used for further analysis with careful data treatment. Although it was difficult to compare the present results to those obtained by previous studies, including studies using the DSI method with a linear combination of docking scores, the comparison of the R and AUC values suggested that the prediction of active compounds by the present models should be comparable to that achieved in the previous study. However, considering the difference of the datasets, the method introduced in the present study should be superior to the previous method. In addition, the present method affords *Δ*G prediction.

In the present study, the docking scores against target proteins were omitted. The QSAR models could be improved by the addition of the docking scores that were descriptors of the models. Thus, the present models should be effective for the correction of docking scores.

## Supporting Information

The compound structures in SDF format and experimental assay data were supplied as described in the supporting information.

## Abbreviations

QSAR: quantitative structure‐activity relationship
DSI: docking score index
MSM: machine‐learning score modification
MTS: multiple target screening
LOO: leave‐one‐out
PCA: principal component analysis
PCR: principal component regression
MLR: multilinear regression
AUC: area under the curve
ROC: receiver operating characteristic

## Appendix A

Suppose f (={f1, f2, f3,…..}) is a set of all pharmacophores. Each pharmacophore (fi) is virtual and the total number of pharmacophores is infinite. Both the protein pocket and compound can be described by the pharmacophore. Each protein pocket p and compound c is projected into the pharmacophore space. The vector product of c1*c2 for compounds c1 and c2 gives the similarity between the compounds, and the vector product p1*p2 for pockets p1 and p2 should give the similarity between the pockets. Since c and p exist in the same space, the vector product p*c corresponds to the similarity between the compound c and pocket p, and it could correspond to the docking score.

## Conflict of Interest

None declared.

## Supporting information

As a service to our authors and readers, this journal provides supporting information supplied by the authors. Such materials are peer reviewed and may be re‐organized for online delivery, but are not copy‐edited or typeset. Technical support issues arising from supporting information (other than missing files) should be addressed to the authors.

Supplementary

Supplementary

## References

[1] Y. Yang , S. J. Adelstein , A. I. Kassis , Drug Discov. Today. 2009, 14, 147–154. 19135549 10.1016/j.drudis.2008.12.005

[2] C. Wermuth , J. Med. Chem. 2004, 47, 1304–1314.

[3] C. Wermuth , Drug Discov. Today. 2006, 11, 160–164. 16533714 10.1016/S1359-6446(05)03686-X

[4] J. H. Nettles , J. L. Jenkins , A. Bender , Z. Deng , J. W. Davies , M. Glick , J. Med. Chem. 2006, 49, 6802–6810. 17154510 10.1021/jm060902w

[5] M. G. Nidhi, M. Glick, J. W. Davis, J. L. Jenkins, *J. Chem. Inf. Model* **2006**, *46*, 1124–1133. 10.1021/ci060003g 16711732

[6] Y. Fukunishi , S. Kubota , H. Nakamura , J. Chem. Inf. Model. 2006, 46, 2071–2084. 16995738 10.1021/ci060152z

[7] Y. Fukunishi , S. Hojo , H. Nakamura , J. Chem. Inf. Comput. Sci. 2006, 46, 2610–2622. 10.1021/ci600334u 17125201

[8] R. P. Sheridan , K. Nam , V. N. Maiorov , D. R. McMasters , W. D. Cornell , J. Chem. Inf. Model. 2009, 49, 1974–1985. 19639957 10.1021/ci900176y

[9] H. Yabuuchi , S. Niijima , H. Takematsu , T. Ida , T. Hirokawa , T. Hara , T. Ogawa , Y. Minowa , G. Tsujimoto , Y. Okuno , Molecular Systems Biology. 2011, 7, 1–12. 10.1038/msb.2011.5 PMC3094066 21364574

[10] S. Niijima , A. Shiraishi , Y. Okuno , J. Chem. Inf. Model. 2012, 52, 901–912. 22414491 10.1021/ci200607f

[11] E. Lounkine , M. J. Keiser , S. Whitebread , D. Mikhailov , J. Hamon , J. L. Jenkins , P. Lavan , E. Weber , A. K. Doak , S. Côté , B. K. Shoichet , L. Urban , Nature 2012, 486, 361–367. 22722194 10.1038/nature11159 PMC3383642

[12] A. Peragovics , Z. Simon , I. Brandhuber , B. Jelinek , P. Hari , C. Hetenyi , P. Czobor , A. Malnasi-Csizmadia , J. Chem. Inf. Model. 2012, 52, 1733–1744. 22697495 10.1021/ci3001056

[13] Z. Simon , A. Peragovics , M. Vigh-Smeller , G. Csukly , L. Tombor , Z. Yang , G. Zahoranszky-Kohalmi , L. Vegner , B. Jelinek , P. Hari , C. Hetenyi , I. Bitter , P. Czobor , A. Malnasi-Csizmadia , J. Chem. Inf. Model. 2012, 52, 134–145. 22098080 10.1021/ci2002022

[14] M. C. Cobanoglu , C. Liu , F. Hu , Z. N. Oltvai , I. Bahar , J. Chem. Inf. Model. 2013, 53, 3399–3409. 24289468 10.1021/ci400219z PMC3871285

[15] A. Peragovics , Z. Simon , L. Tombor , B. Jelinek , P. Hari , P. Czobor , A. Malnasi-Csizmadia , J. Chem. Inf. Model. 2013, 53, 103–113. 23215025 10.1021/ci3004489

[16] V. I. Perez-Nueno , D. W. Ritchie , J. Chem. Inf. Model. 2011, 51, 1233–1248. 21604699 10.1021/ci100492r

[17] V. I. Perez-Nueno , A. S. Karaboga , M. Souchet , D. W. Ritchie , J. Chem. Inf. Model. 2014, 54, 720–734. 24494653 10.1021/ci4006723

[18] H. B. Engin , O. Keskin , R. Nussinov , A. Gursoy , J. Chem. Inf. Model. 2012, 52, 2273–2286. 22817115 10.1021/ci300072q PMC3979525

[19] Y. Pan , T. Chemg , Y. Wang , S. H. Bryant , J. Chem. Inf. Model. 2014, 54, 407–418. 24460210 10.1021/ci4005354 PMC3956470

[20] J. Tang , A. Szwajda , S. Shakyawar , T. Xu , P. Hintsanen , K. Wennerberg , T. Aittokallio , J. Chem. Inf. Model. 2014, 54, 735–743. 24521231 10.1021/ci400709d

[21] R. P. Sheridan , Chem. Inf. Model. 2014, 54, 1083–1092. 10.1021/ci500084w 24628044

[22] M. Lindh , F. Svensson , W. Schaal , J. Zhang , C. Skold , P. Brandt , A. Karlen , J. Chem. Inf. Model. 2015, 50, 343–353. 10.1021/ci5005465 25564966

[23] Y. Wang , J. Xiao , T. O. Suzek , J. Zhang , S. H. Bryant , Nucleic Acids Res. 2009, 37, W623–W633. 10.1093/nar/gkp456 PMC2703903 19498078

[24] A. Gaulton , L. J. Bellis , A. P. Bentro , J. Chambers , M. Davies , A. Hersey , Y. Light , S. McGlinchey , D. Michalovich , B. Al-Lazikani , P. Overington , Nucleic Acids Res. 2011, 40, D1100–D1107. 10.1093/nar/gkr777 PMC3245175 21948594

[25] O. P. J. van Linden , A. J. Kooistra , R. Leurs , I. P. de Esch , C. de Graal , J. Med. Chem. 2014, 57, 249–277. 23941661 10.1021/jm400378w

[26] T. Anastassiadis , S. W. Deacon , K. Devarajan , H. Ma , J. Peterson , Nature Biotechnology. 2011, 29, 1039–1046. 10.1038/nbt.2017 PMC3230241 22037377

[27] M. I. Davis , J. P. Hunt , S. Herrgard , P. Ciceri , L. M. Wodicka , G. Pallares , M. Hocker , D. K. Treiber , P. P. Zarrinkar , Nature Biotechnology. 2011, 29, 1046–1052. 10.1038/nbt.1990 22037378

[28] Y. Fukunishi , Y. Mikami , K. Takedomi , M. Yamanouchi , H. Shima , H. Nakamura , J. Med. Chem. 2006, 49, 523–533. 16420039 10.1021/jm050480a

[29] Y. Fukunishi , S. Kubota , H. Nakamura , J. Mol. Graph. Model. 2007, 25, 633–643. 16777448 10.1016/j.jmgm.2006.05.001

[30] M. Masuda , H. Kaneko , K. Funatsu , Ind. Eng. Chem. Res. 2014, 53, 8553–8564.

[31] Y. Fukunishi , Y. Mikami , H. Nakamura , J. Mol. Graph. Model. 2005, 24, 34–45. 15950507 10.1016/j.jmgm.2005.04.004

[32] F. P. Steinmetz , J. C. Madden , M. T. D. Cronin , J. Chem. Inf. Model. 2015, 55, 1739–1746. 26186603 10.1021/acs.jcim.5b00294

[33] I. Cortes-Ciriano , A. Bender , J. Chem. Inf. Model. 2015, 55, 2682–2692. 26619900 10.1021/acs.jcim.5b00570

[34] Y. Cheng , W. H. Prusoff , Biochem. Pharmacol. 1973, 22, 3099–3108. 4202581 10.1016/0006-2952(73)90196-2

[35] D. Kitagawa , K. Yokota , M. Gouda , Y. Narumi , H. Ohmoto , E. Nishiwaki , K. Akita , Y. Kirii , Genes Cells. 2013, 18, 110–1022. 23279183 10.1111/gtc.12022

[36] Z. A. Knight , K. M. Shokat , Chem. Biol. 2005, 12, 621–637. 15975507 10.1016/j.chembiol.2005.04.011

[37] I. Kufareva , R. Abagyan , J. Med. Chem. 2008, 51, 7921–7932. 19053777 10.1021/jm8010299 PMC2669721

[38] D. A. Case, T. A. Darden, T. E. Cheatham III, C. L. Simmerling, J. Wang, R. E. Duke, R. Luo, K. M. Merz, B. Wang, D. A. Pearlman, M. Crowley, S. Brozell, V. Tsui, H. Gohlke, J. Mongan, V. Hornak, G. Cui, P. Beroza, C. Schafmeister, J. W. Caldwell, W. S. Ross, P. A. Kollman, AMBER 8, UCSF, **2004**.

[39] Y. Fukunishi , Y. Mikami , H. Nakamura , J. Phys. Chem. B. 2003, 107, 13201–13210.

[40] J. Wang , R. M. Wolf , J. W. Caldwell , P. A. Kollman , D. A. Case , J. Compt. Chem. 2004, 25, 1157–1174. 15116359 10.1002/jcc.20035

