# Supplementary material for: Quantitative Structure‐activity Relationship (QSAR) Models for Docking Score Correction
Source: Mol Inform. 2016 Apr 29;36(1-2):1600013. doi: 10.1002/minf.201600013 (PMC5297997; doi:10.1002/minf.201600013)
Supplement: Supplementary file 1 — Supplementary [file MINF-36-0-s001.pdf]

molecular informatics
models – molecules – systems

Supporting Information

APPENDIX B

The Sievgene score is determined as

$$S_{\Delta G} = c_{rot} \cdot N_{rot} + c_{AV} \cdot (E_{ASA} + E_{vdW}) + c_{ele} \cdot E_{ele} + c_{hyd} \cdot E_{hyd} + c_{intra-vdW} \cdot E_{intra-vdW},$$

where N_{rot}, E_{ASA}, E_{vdW}, E_{ele}, E_{hyd} and $E_{intra\text{-}vdW}$ represent the number of rotatable bonds of the docked compound, the hydrophobic energy due to the accessible surface area, the van der Waals energy, the protein-ligand Coulomb potential, the hydrogen bond energy and the intramolecular vdW energy of the ligand. In addition, c_{rot}, c_{AV}, c_{ele}, c_{hyd} and $c_{intra\text{-}vdW}$ are the optimized coefficients for each energy term. For each atom type, the sum of E_{ASA} and E_{vdW} gives one grid potential. Sievgene utilizes the grid potential to calculate each energy term except the intramolecular interaction.

APPENDIX C

The selected 600 proteins were as follows: 10gs, 13gs, 17gs, 19gs, 1a0l, 1a27, 1a3k, 1a5h, 1aax, 1ad5, 1ae8, 1afe, 1agw, 1aht, 1aq1, 1aqw, 1aqx, 1atk, 1au0, 1au2, 1au3, 1au4, 1avn, 1awf, 1awh, 1axs, 1ayu, 1ayv, 1ayw, 1b09, 1b2y, 1b38, 1b39, 1b3d, 1b55, 1bhx, 1bic, 1bik, 1bio, 1biw, 1bj4, 1bkf, 1bl4, 1bl6, 1bl7, 1bmk, 1bmq, 1bpy, 1bui, 1bwn, 1byg, 1bzc, 1bzj, 1bzm, 1bzs, 1bzy, 1c1u, 1c1v, 1c1y, 1c4u, 1c4y, 1c5c, 1c5o, 1c5w, 1c5x, 1c5y, 1c8t, 1c9h, 1c9y, 1ca8, 1cam, 1can, 1caq, 1cay, 1caz, 1cbq, 1cbs, 1cc0, 1cd9, 1cea, 1ceb, 1ciz, 1ckp, 1cqp, 1ctr, 1d4p, 1d4x, 1d5j, 1d5m, 1d6v, 1d7h, 1d7i, 1d7j, 1d7x, 1d8f, 1db1, 1db4, 1db5, 1dcy, 1dhf, 1dhs, 1di8, 1di9, 1doa, 1doj, 1drf, 1dtw, 1dvs, 1dvt, 1dvu, 1dvx, 1dvy, 1dvz, 1dwb, 1dwc, 1dwd, 1e1v, 1e1x, 1e1z, 1e2d, 1e2q, 1e2s, 1e3c, 1e3k, 1e4h, 1e51, 1e5a, 1e7a, 1e7b, 1e7c, 1e7e, 1e7f, 1e7g, 1e7h, 1e7i, 1e87, 1e96, 1e9h, 1eax, 1ejn, 1ek5, 1ekf, 1ekp, 1ekv, 1emu, 1eou, 1ep9, 1equ, 1ere, 1err, 1exa, 1exx, 1f0r, 1f2w, 1f45, 1f5f, 1f5n, 1f9p, 1fak, 1fao, 1fby, 1fcx, 1fcy, 1fcz, 1fd0, 1fds, 1fdt, 1fdw, 1fgi, 1fhi, 1fin, 1fjs, 1fkb, 1fkd, 1fkf, 1fkg, 1fkh, 1fki, 1fkj, 1fls, 1fpc, 1fq1, 1fuj, 1fvo, 1fzc, 1fze, 1fzf, 1fzg, 1fzv, 1g05, 1g2l, 1g2m, 1g30, 1g32, 1g3m, 1g49, 1g4k, 1g5s, 1g7f, 1g7g, 1g86, 1g9n, 1gbn, 1gc1, 1gcq, 1gfw, 1gkc, 1gkd, 1gmn, 1gmo, 1gni, 1gos, 1gra, 1gs4, 1gua, 1guv, 1gv7, 1gwq, 1gwr, 1gyk, 1gzp, 1gzq, 1gzr, 1gzy, 1gzz, 1h07, 1h08, 1h0c, 1h0v, 1h0w, 1h1b, 1h1h, 1h1v, 1h1w, 1h2m, 1h2n, 1h2t, 1h2u, 1h52, 1h53, 1h8i, 1h9u, 1he2, 1he7, 1he8, 1hh8, 1hi3, 1hi4, 1hi5, 1i0z, 1i4d, 1i4l, 1i4t, 1i5r, 1i76, 1i7g, 1i7i, 1ibr, 1icf, 1iei, 1ii6, 1iiq, 1iis, 1iix, 1ikt, 1ikx, 1il0, 1iqe, 1iqf, 1iqg, 1iqh, 1iqj, 1iqk, 1iqm, 1iqn, 1ira, 1itq, 1itu, 1ivy, 1j1b, 1j1c, 1j78, 1j8h, 1j8u, 1j96, 1j99, 1jag, 1jbq, 1jbu, 1jdn, 1jdp, 1jdx, 1jgd, 1jiz, 1jj9, 1jk3, 1jk7, 1jk8, 1jkk, 1jqd, 1jqe, 1jsv, 1ju6, 1juj, 1jvp, 1jvq, 1jwh, 1jwt, 1k21, 1k22, 1k27, 1k3l, 1k5d, 1k8r, 1kak, 1kav, 1kbc, 1kcg, 1kdk, 1kfa, 1khb, 1khf, 1kjl, 1kjr, 1kkq, 1klt, 1kmv, 1knu, 1kq0, 1kts, 1ktt, 1kv1, 1kv2, 1kw0, 1kye, 1l2j, 1l8j, 1l8l, 1li4, 1lpk, 1ly2, 1m17, 1m27, 1m2z, 1m3h, 1m49, 1m4a, 1m4b, 1m4u, 1m51, 1m6w, 1m76, 1m7q, 1m9z, 1mc5, 1men, 1mkd, 1mmb, 1mmk, 1mmt, 1moo, 1mq5, 1mq6, 1mrq, 1msv, 1mu2, 1mu6, 1mu7, 1mu8, 1mu9, 1mue, 1mvs, 1mvt, 1mx1, 1mzf, 1n6c, 1n6h, 1n6k, 1n6l, 1n6n, 1n6o, 1n6p, 1n6r, 1n7i, 1n7j, 1n83, 1n86, 1n8z, 1nav, 1nax, 1nb3, 1nb5, 1nbp, 1nde, 1ne7, 1nf3, 1nf7, 1nhx, 1nhz, 1njs, 1nju, 1nkm, 1nl6, 1nl9, 1nlj, 1nlv, 1nm1, 1nm6, 1nmd, 1nmq, 1nmx, 1nmy, 1nmz, 1nn0, 1nn1, 1nn3, 1nn5, 1nn6, 1nny, 1no6, 1no9, 1now, 1np0, 1nrg, 1nrl, 1nsi, 1nt1, 1nun, 1nup, 1nvq, 1nvr, 1nvs, 1nwl, 1nwr, 1nws, 1nwt, 1nwu, 1ny3, 1nyx, 1nz7, 1nzq, 1o0d, 1o0v, 1o41, 1o42, 1o43, 1o44, 1o45, 1o46, 1o47, 1o48, 1o49, 1o4a, 1o4b, 1o4d, 1o4e, 1o4f, 1o4g, 1o4h, 1o4i, 1o4j, 1o4k, 1o4l, 1o4m, 1o4n, 1o4o, 1o4p, 1o4q, 1o4r, 1o5d, 1o5e, 1o5f, 1o6e, 1o6k, 1o6l, 1o6u, 1o86, 1o9s, 1oat, 1oec, 1of7, 1ogt, 1ogu, 1ohj, 1ohk, 1oi9, 1oiq, 1oir, 1oit, 1oiu, 1oiv, 1oiw, 1oix, 1ojb, 1ojc, 1ojd, 1okl, 1okm, 1okn, 1oky, 1okz, 1ol0, 1olm, 1ols, 1olu, 1olx, 1ony, 1onz, 1op3, 1oq5, 1osf, 1oth, 1ouk, 1ouy, 1ov4, 1ove, 1ovz, 1ow0, 1ow3, 1oyn, 1oyt, and 1oz1. For 10gs, 13gs, 17gs, 19gs, 1aax, 1ad5, 1ad8, 1aqw, 1aqx, 1avn, 1bb5, 1bic, 1bio, 1bpy, 1bzs, 1c5x, 1e2s, 1e7a, 1e7b, 1e7c, 1exa, 1exx, 1f5k, 1fak, 1fcx, 1fcy, 1fcz, 1fd0, 1fdt, 1fjs, 1fzc, 1g3m, 1g4k, 1g86, 1gc1, 1gni, 1gra, 1gzp, 1gzq, 1h07, 1h08x3, 1h0cx3, 1i5r, 1iei, 1ii6, 1itu, 1j78, 1jdp, 1li4, 1mc5, 1mrq, 1nrg, 1op3, and 1op3. For some proteins, two pockets were prepared, since each of these proteins binds two ligands.
